# Electrochemical and X-ray Photoelectron Spectroscopic Study of Early SEI Formation and Evolution on Si and Si@C Nanoparticle-Based Electrodes

**DOI:** 10.3390/ma15227990

**Published:** 2022-11-11

**Authors:** Antoine Desrues, Eric De Vito, Florent Boismain, John P. Alper, Cédric Haon, Nathalie Herlin-Boime, Sylvain Franger

**Affiliations:** 1 NIMBE, CEA, CNRS, Université Paris-Saclay, 91191 Gif-sur-Yvette, France; 2 Université Grenoble Alpes, CEA, Liten, DTNM, 38000 Grenoble, France; 3 Université Grenoble Alpes, CEA, Liten, DEHT, 38000 Grenoble, France; 4 ICMMO, UMR CNRS 8182, Université Paris-Saclay, 91405 Orsay, France

**Keywords:** silicon, battery, Electrochemical Impedance Spectroscopy (EIS), X-ray Photoelectron Spectroscopy (XPS)

## Abstract

Carbon coatings can help to stabilize the electrochemical performance of high-energy anodes using silicon nanoparticles as the active material. In this work, the comparison of the behavior and chemical composition of the Solid Electrolyte Interphase (SEI) was carried out between Si nanoparticles and carbon-coated Si nanoparticles (Si@C). A combination of two complementary analytical techniques, Electrochemical Impedance Spectroscopy and X-ray Photoelectron Spectroscopy (XPS), was used to determine the intrinsic characteristics of the SEI. It was demonstrated that the SEI on Si particles is more resistive than the SEI on the Si@C particles. XPS demonstrated that the interface on the Si particles contains more oxygen when not covered with carbon, which shows that a protective layer of carbon helps to reduce the number of inorganic components, leading to more resistive SEI. The combination of those two analytical techniques is implemented to highlight the features and evolution of interfaces in different battery technologies.

## 1. Introduction

Developing safe, stable and long-life electrochemical energy storage devices is one key to achieving the energy transition, a major challenge of the 21st century. Lithium-ion technology is currently widely commercialized for electronic devices and electric vehicles owing to its high power and energy capabilities [1]. Graphite is the most common negative electrode used in such devices due to its high cyclability, low cost and non-toxicity. Its reversible capacity is 372 mAh/g, obtained through a lithium intercalation–deintercalation mechanism in the lamellar structure of the graphite [2].

Significant effort has been put toward improving the energy density of Li-ion batteries. Among negative active materials, the best candidate so far is silicon, which can exceed the capacity of graphite by up to ten times (up to 3579 mAh/g) by forming Li_x_Si alloys. Moreover, silicon is the second most abundant element in the earth’s crust, which makes it a low-cost raw material and it is also non-toxic. Its low operating voltage of 0.4 V vs. Li^+^/Li enables high energy density applications [3]. However, silicon suffers from a volumetric expansion and contraction of 320% during lithiation and delithiation, which leads to severe mechanical stress and instability [4]. Actually, this can induce, during cycling, electrode delamination, cracking and loss of electronic percolation. All these events will lead to rapid capacity fade [5]. The volume expansion also damages the Solid Electrolyte Interphase (SEI), which is formed at low potential by the degradation products from the liquid electrolyte [6]. The SEI is known to be an efficient protective buffer layer, especially on anode electrodes [7,8], leading to better chemical stability between the electrolyte and the electrode at lower voltages. However, its proper formation at the very early stages of the battery life (such as homogeneity, thickness, and porosity) and its mechanical stability upon cycling (adherence) are prominent key factors for long-lasting performance. In the case of silicon-based anodes, the subsequent volumetric variations will progressively fracture the SEI, inducing a constant exposition of bare silicon surfaces, where new SEI can be deposited. This excessive generation/dissolution of SEI will be finally responsible for porosity filling and particle insulation, also leading to capacity fade and poor Coulombic efficiency [9].

Several strategies have then been developed to solve those issues: the use of submicronic silicon particles (particle diameter < 150 nm) allows for avoiding primary fractures of the active material, while the development of carbon-coated silicon allows the formation of a more stable SEI (considering the carbon surface is less chemically reactive than silicon) [10,11,12,13,14,15].

A variety of silicon core–carbon shell nanostructures were thus designed, as those nanostructures are the best candidates so far. However, they generally require multiple synthesis steps. The role of the carbon coating in enhancing the electronic percolation [14] is now well understood, whereas the noticeable differences in SEI properties deposited either on silicon or carbon surfaces remain unclear, as well as the SEI evolution during the cycling.

The Si@C nanoparticles, presented in this work, consisting of a silicon core embedded in a carbon shell. They were synthesized with a double-stage laser pyrolysis setup, through a one-step gas phase technique developed in the lab that was previously described [16,17]. Moreover, the protection of the silicon core by the carbon shell also prevents the formation of a silicon oxide layer, which was proven to impact both the structure and the composition of the SEI to be formed [18,19].

SEI chemical composition and formation dynamics can actually be modified by changing the surface properties. Several indirect parameters could impact the different properties of the SEI, such as its resistance, its capacitance or its charge transfer resistance. Electrochemical Impedance Spectroscopy (EIS) can provide these pieces of information. Impedance analysis of SEI was already carried out. Some authors have followed the changes in the characteristic semi-circle associated with SEI [14], whereas others have performed deeper investigations by analyzing the evolution of both the resistance and the capacitance of the SEI for Si and Si@C materials [20]. To help in the understanding of EIS results by identifying the SEI chemical composition changes, X-ray photoelectron spectroscopy (XPS) is commonly used such as for the characterization of lithium alkyl carbonates [21] or the role of LiPF_6_ [22] for Li-ion anodes.

Herein, the study of the SEI evolution on pure silicon and on carbon-coated silicon particles during the first cycle is presented. An original combination of two analytical techniques, Electrochemical Impedance Spectroscopy (EIS) and X-ray Photoelectron spectroscopy (XPS), was successfully applied. This work allowed us to shed more light on the beneficial impact of the carbon coating on the Si@C nanoparticles.

## 2. Results and Discussion

### 2.1. Material Characterization

Two samples of crystalline-based silicon particles were synthesized. One is a silicon core of 30 nm diameter and the second is composed of the same core, with a carbon coating. More specifically, the carbon coating exhibits a 2 nm-thickness, which represents 19 wt% of the total mass of the particles. Those diameters are calculated from the BET (Brunauer–Emmet–Tellet) surface analysis, assuming that particles are spherical. The measured BET surfaces were 89 and 92 m^2^/g for Si and Si@C materials, respectively.

**Figure 1:**
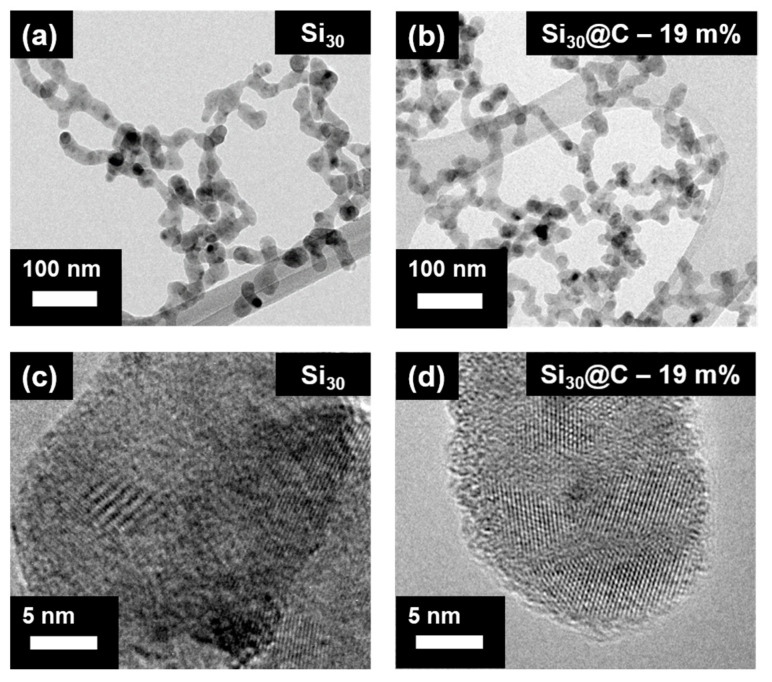
TEM micrographs of Si (**a**) and Si@C (**b**) nanoparticles where the typical chain-like morphology can be observed. (**c**,**d**) represent the same materials at a higher magnification. A shell of amorphous carbon is observed at the edge of the Si@C particles on (**d**).

The particle shapes can be observed in Figure 1, with the classical chain-like morphologies of particles made by laser pyrolysis. As previously described in [16,17], laser pyrolysis leads to narrow-size particle distribution. The XRD (X-ray Diffraction) shows the crystallinity of the silicon active material and the Raman spectrum of carbon confirms its disordered nature (Figure S1a,b), which can be also confirmed by the HRTEM (High-Resolution Transmission Microscopy) micrographs in Figure 1b. Actually, the carbon shell is mostly amorphous, as can be observed with the D and G bands in the Raman spectroscopy. It is important to observe that no SiC can be detected by XRD and no carbonaceous particles are observed (Figure S1a).

**Figure 2:**
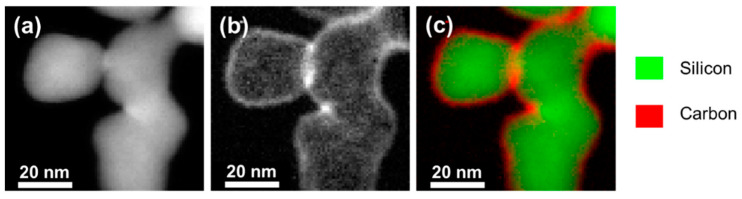
STEM–EELS (pictures of the Si@C materials). (**a**) silicon signal, (**b**) carbon signal, (**c**) superposition of the two signals. Si is depicted in green and C in red. The core-shell structure of the material is demonstrated.

STEM–EELS (Scanning Transmission Electron microscopy–Electron Energy Loss spectroscopy) experiments were carried out and are presented in Figure 2. The electrons emitted from the silicon are presented in Figure 2a, whereas the ones emitted by the carbon are presented in Figure 2b. Figure 2c clearly demonstrates that the core-shell structure is achieved. The carbon shell is homogeneous and the thickness of the deposit is around 2 nm. The total mass of carbon is assumed to be only located around the Si cores, forming a powder batch with only the silicon-core/carbon-shell nanoparticles.

### 2.2. Electrochemical Performance

**Figure 3:**
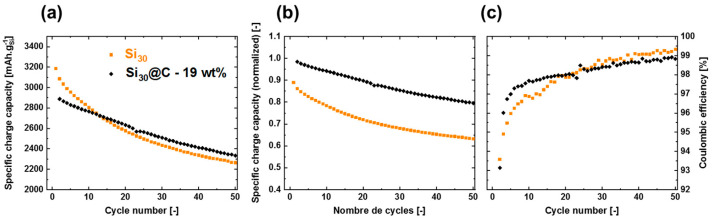
(**a**) Galvanostatic cycling results of both studied materials Si_30_ and Si_30_@C—19 wt% over the first 50 cycles (**b**) normalized galvanostatic results for the same materials. The specific capacity at each cycle is divided by the charge capacity at the first cycle, taking into account the specific capacity of carbon at 150 mAh·g^−1^. (**c**) Coulombic efficiency for the same materials.

The electrochemical performance of the two different materials was tested to highlight the effect of the carbon shell. The specific charge capacity (in mAh/g_Si_) for both materials is presented in Figure 3a. They were recorded at a C/5 rate. The corresponding capacities gradually decrease but the capacity fade of Si is more important, especially during the first cycles.

The capacities were normalized to take into account the capacity of the carbon in Si@C. It was shown that the capacity of carbon, synthesized through laser pyrolysis, was 150 mAh/g [23]. Assuming the total activity of the carbon (19%) has a specific capacity of 150 mAh/g, whereas 81% of the powder (silicon) has a specific capacity of 3579 mAh/g, the specific capacity of Si@C nanoparticles is thus 2935 mAh/g. Normalized results are shown in Figure 3b and prove the stabilizing effect of the carbon shell compared to the bare material surfaces. This advantageous effect can be observed at the early stage of the first cycles, as shown by the better Coulombic efficiencies in the case of Si@C for cycles 3 to 20 (Figure 3c).

For silicon-based materials, the low Coulombic efficiency is related to the important SEI formation. The only difference between both materials is the presence of the carbon shell. Thus, such important differences in the SEI formation and evolution can be attributed to the surface properties and especially the distinct reactivity between Si and C, which can lead to different SEI chemistries (more or less organic) and morphologies (more or less porous). It was thus decided to study the formation and the evolution of the SEI on both materials by coupling analyses with Electrochemical Impedance Spectroscopy (EIS) and X-ray Photoelectron spectroscopy (XPS).

### 2.3. Impedance Measurements (EIS)

**Figure 4:**
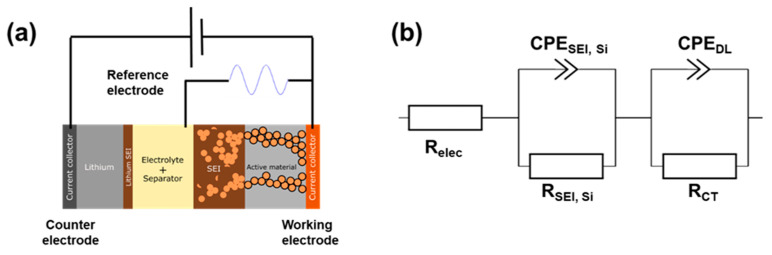
(**a**) Scheme of the cell in a three electrodes configuration; (**b**) the corresponding equivalent electrical circuit to model and fit raw data from measurement.

EIS measurements were performed with a three-electrode cell setup. A scheme of this configuration is shown in Figure 4a. The advantage of using a reference electrode is that the impedance collected can be easily assigned to the sole working electrode side (i.e., the electrified interface of the silicon-based electrode studied here). As this work was focused on the SEI formation/deposition, only the data obtained at high frequency were assigned and interpreted. Actually, the kHz domain corresponds to the interface phenomena since the wavelength is too short to penetrate the bulk electrode. Keeping in mind this specific frequency range, the equivalent electrical circuit to model and fit the data of interest for this study is presented in Figure 4b.

The first R//C circuit is used to model the resistance of the SEI deposit and its capacitance and the second R//C circuit model the charge transfer resistance and the double-layer capacitance. Those two capacitances are not modeled by ideal capacitors since the surface of the electrode is not planar, due to the architecture of the composite electrode. Indeed, capacitive phenomena are here modeled by constant phase elements (CPE), where a deviation parameter alpha is added to take into account the roughness and/or inhomogeneity of the exposed surfaces. The value of alpha should remain, for physical meaning, between 0.7 and 1 (with 1 standing for an ideal capacitor).

**Figure 5:**
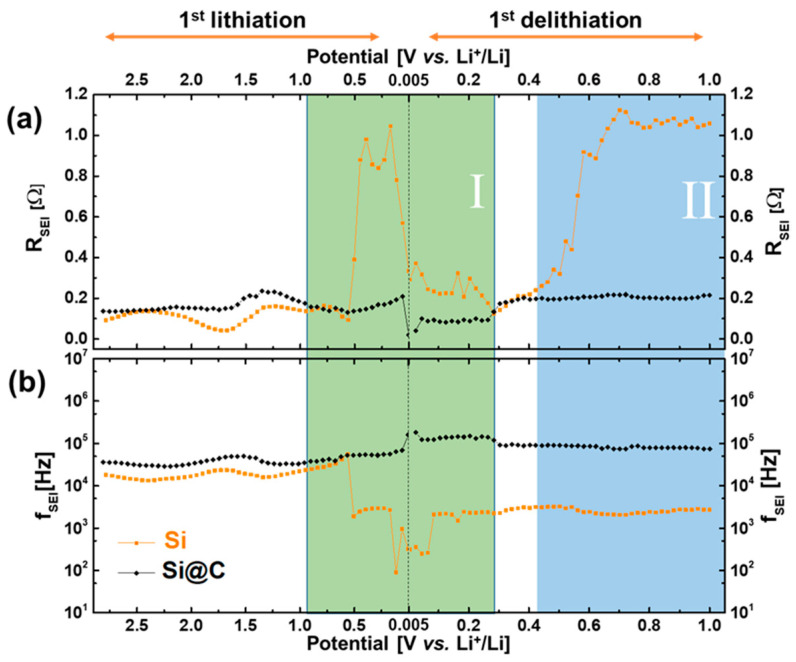
(**a**) Resistances and (**b**) frequencies of the SEI for both Si (orange) and Si@C (black) materials. Two distinct zones exhibit different behaviors of resistances and/or frequencies.

The technique used to probe the impedance is a potentially resolved EIS. After an initial EIS measurement (at OCV), different potential steps are applied to the electrode. Fifty-one measurements were carried out, with fixed intervals (SI-2). This protocol allows the measurement of the impedance along the first lithiation by bringing the Si electrode progressively to a discharged state and then along the first delithiation. The evolution of the overall impedance can thus be probed. Fitting of each individual EIS spectrum is then carried out with the equivalent model in order to extract the different values of resistance and capacitance, especially for the SEI. Those data are essential parameters that characterize the SEI. Resistances of the SEI for Si and Si@C during the lithiation and delithiation are presented in Figure 5a. The resistance evolution, during the first cycle, for both materials, can thus be compared.

An increase in resistance can be the signature of two distinct phenomena, which need to be differentiated: (i) an increase in the SEI thickness (leading to a slowdown of the mobility of the ions), (ii) a change in the SEI chemical composition (deposition of different (in)organic species can change the conductivity of the layer). Following the relaxation frequency of the SEI allows us to unambiguously discriminate between both phenomena. Actually, this frequency is expressed by the following formula:

$$f_{SEI,Si}=\frac{1}{2\pi \;R_{SEI,Si}\;C_{SEI,Si}}$$

which can be simplified as:

$$f_{SEI,Si}=\frac{\sigma_{SEI,Si}}{2\pi \;\varepsilon_{0}\;\varepsilon_{r}}$$

In this latter formula, all the parameters (*σ_SEI,Si_*, *ε*_0_, *ε_r_*) are only related to the chemical composition of the SEI (i.e., its intrinsic physical properties). Indeed, geometrical parameters (thickness, surface) of the SEI do not appear in the frequency expression.

In Figure 5a, two behaviors concerning resistance evolution can be observed. The R_SEI,Si_ on the Si@C particles (black data) is globally stable around 0.2 Ω, except for a slight drop at the very beginning of delithiation. The R_SEI,Si_ on the Si particles (orange data) evolves much more during the voltage sweep. Around 0.5 V (lithiation), the SEI resistance increases by a factor of 9 (from 0.1 to 0.9 Ω). It then decreases to its original value, before reaching the final reduction potential of 0.005 V (lithiation) and increases again (by the same factor of 9) after 0.4 V (delithiation). At the end of the delithiation process (1 V), the R_SEI,Si_ has thus increased only in the case of the Si particles.

Relaxation frequencies (Figure 5b) also exhibit different behaviors depending on the material tested. The *f_SEI,Si_* of Si@C particles (black data) remains almost stable, all along the first lithiation and delithiation processes, at a value around 10^5^ Hz. The *f_SEI,Si_* evolution for Si particles (orange data) is, here again, more important. Frequency values are first (lithiation) around 10^4^ Hz and then fall down and stabilize at 10^3^ Hz (between 0.5 and 0.2 V) before decreasing again around 5.10^2^ Hz (end of lithiation, beginning of delithiation). These differences are significant enough and highlight that the SEI on the Si particles becomes globally more resistive, all over this voltage window. Above 0.1 V (delithiation), the frequency value again reaches 10^3^ Hz and remains constant until 1 V, consistent with a slightly more conductive SEI.

Two zones can then be determined for Si nanoparticles, where a difference in both the resistance and the frequency behavior can be observed. They are represented in green (Zone I) and in blue (Zone II) in Figure 5a,b. The drop in the frequency in Zone I can be attributed to a change in the SEI chemical composition (more resistive products). A concomitant increase in the SEI thickness (and its corresponding R_SEI,Si_) could remain a possibility at this stage. On the contrary, in Zone II, we only observe an increase in R_SEI,Si_ and no change in the frequency, which is the signature of a thicker SEI deposition of the same composition. EIS measurements thus evidenced that the deposited SEI on bare Si nanoparticles has noticeably different chemical compositions upon voltage sweep, whereas its nature remains almost identical for Si@C nanoparticles, all along the first cycle. Moreover, it can also be predicted that the SEI chemical composition is different for both silicon-based materials since the respective SEI relaxation frequencies exhibit a difference of one order of magnitude.

In order to gain some more valuable information on both the stability and the impedance of the deposited SEI on the different silicon-based materials, 50 cycles of successive charge–discharges at a C/5 rate were performed.

**Figure 6:**
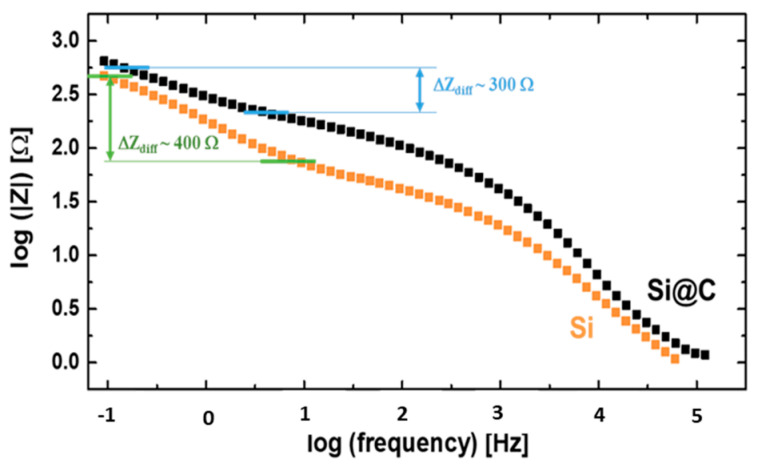
EIS Bode plots after 50 cycles, enlightening the diffusion impedance domains for both Si (orange square) and Si@C (black dot) materials.

Figure 6 shows the Bode diagrams (Log*|Z|* as a function of Log *f*) for both the Si and Si@C materials after 50 cycles. It is interesting to observe that the impedance related to the lithium diffusion process in the electrode is more important in the case of the Si nanoparticles (~400 Ω) compared to Si@C (~300 Ω). This could be due to at least two factors, previously evidenced in the first cycle: (i) for the Si material, the SEI can be partially dissolved/removed at each end of lithiation/beginning of delithiation, and (ii) the SEI can become densified upon charging.

Once again, the presence of the carbon coating around the silicon nanoparticles to lower (or delay) the SEI evolution seems crucial: this prevents the porosities of the electrode from being rapidly filled by SEI by-products detached from the surface, and leading progressively to the electrode asphyxia, as evidenced in [24].

### 2.4. Chemical Analysis by X-ray Photoelectron Spectroscopy

At this point, XPS experiments were carried out to better understand the evolutions observed in EIS electrical equivalent parameters. The samples were chosen at six different voltage values during the first cycle of lithiation and delithiation. An electrode wetted by the electrolyte was also analyzed before cycling as a reference to check the surface chemical composition before any voltage change.

**Figure 7:**
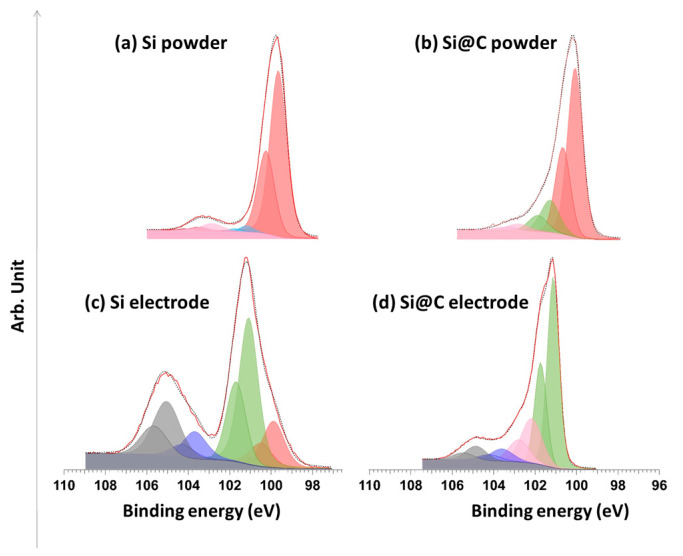
Si 2p XPS spectra for (**a**) pristine Si powder, for (**b**) pristine Si@C powder, for both materials embedded inside a composite electrode with (**c**) Si and for (**d**) Si@C.

A comparison of the Si 2p spectra for pristine Si and pristine Si@C nanoparticles is presented in Figure 7a,b. Table 1 summarizes the contributions which can be generally observed for silicon anodes. As expected, both spectra display an important contribution to Si^0^. Silicon suboxides are detected in small quantities (doublets located within the [101.0; 102.5] eV range.

Figure 7c,d show the silicon signal after both powders were prepared in a slurry, cast on the electrodes, assembled in a coin cell, and exposed to the electrolyte at open circuit voltage. The differences between Figure 7a,c,d can be explained (i) by the suspension of the powder in water (during the slurry preparation) and (ii) by the ulterior exposition of the electrolyte. For Si nanoparticles, those preparation steps lead to an intense doublet at 101.2 eV (Si suboxides or Si-O-C bonds), other doublets related to Si suboxides in the [101.0; 103.0] eV range, a doublet at 103.7 eV (SiO_2_) and another one at 105 eV (Si-F). The two reasons behind this are: (i) the exposure to water leads to the formation of an oxide layer (and the appearance of Si-O bonds), (ii) the mixing with the CMC binder can lead to the formation of Si-O-C bonds, and (iii) the presence of FEC can lead to the formation of O-Si-F bonds, as described in [22].

Himpsel et al. [28] demonstrated that the shift of the silicon oxide peak towards higher energies was proportional to the oxygen quantity “x” in SiO_x_ samples. Assuming that the quantity x of oxygen varies linearly between E_Si_ = 99.5 eV and E_SiO2_ = 103.7 eV, with a shift of 1.8 eV, as observed here, the average composition of our oxide layer would be SiO_0.85_.

The existence of a peak assigned to Si-O-C was first reported by Ekoué et al. [30]. The corresponding energy is at 101.3 eV. Those bonds can be formed by the condensation of silanol chemical function (Si-OH), present at the surface of the non-carbon-coated nanoparticles, with the alcohol chemical function of the CMC. The CMC used here is methylated at 70%, allowing the other 30% free OH groups to condensate.

Figure 7d presents the Si 2p peak of the Si@C active material in the electrode. A doublet is observed at 101.2 eV and can be attributed to SiOx and Si-O-C bonds. The formulation step may also lead to the supplementary formation of Si-C bonds due to the high temperature of the drying step under vacuum (48 h at 180 °C). Three other doublets at energies of [102.2 eV; 102.8 eV], [103.7 eV; 104.2 eV] and [105.0 eV; 105.6 eV] correspond to different oxidation states of the silicon, as reported in Table 1. The latter is attributed to O-Si-F bonds, whereas the two first ones are, respectively, attributed to Si suboxides and SiO_2_ oxides. O-Si-F bonds are related to the breaking of Si-O bonds by HF appearing with the degradation of FEC molecules [31]. Moreover, the carbon coating appears to protect the silicon surface from oxidation since the Si^0^/Si^4+^ intensity ratio is three times bigger for the Si@C nanoparticles.

Exposure to electrolytes has a strong impact on Si and Si@C nanoparticle surfaces, especially on the thicknesses of the surface layers. Indeed, the Si^0^ signal is strongly attenuated (for Si) and even disappears (for Si@C), which suggests a thicker deposit and a more important SEI formation (Figure 7). Moreover, because Si is “protected” by the carbon coating, it appears that the formation of Si-O-C bonds is favored, hindering the formation of Si-O-F bonds.

**Figure 8:**
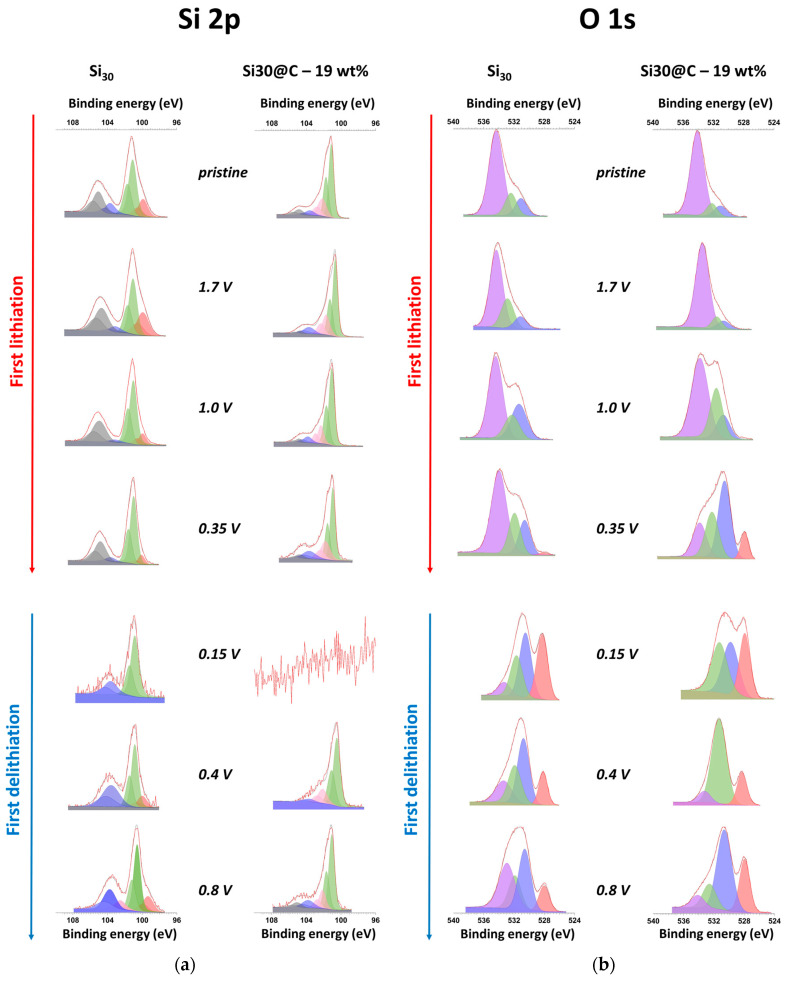
Example of deconvolution in different contributions of Si 2p (**a**) and O 1s (**b**) XPS signals. Each color represents the same contribution.

The XPS measurements were achieved on six different orbitals (Si 2p, O 1s, C 1s, Li 1s, F 1s, P 2p), which are representative of the SEI. As an example, Figure 8 shows the evolution of the high-resolution spectra obtained for the Si 2p and O 1s orbitals all along the first cycle. Quantification was performed by deconvolution of each peak as several contributions. The atomic percentage of each element in each binding configuration can thus be obtained. Some of the useful information can be summarized to shed more light on the SEI structure.

Figure 8a displays the evolution of the silicon signal for both materials. The major difference between Si and Si@C nanoparticles is linked with Si^0^. The decrease and vanishing of this peak, in the case of Si, is attributed to the SEI thickening, as previously suggested by the impedance study. This contribution appears again at 0.8 V during delithiation, which is a sign of a thinner SEI. This contribution is never observed for Si@C because the thickness of the deposited layer is too high (>6 nm, the detection limit for XPS surface analysis for the chosen experimental configuration).

The quantity of SEI formed is higher when the voltage is close to 5 mV, most probably because of the organic solvent’s instability. For Si, the experimental spectrum is noisy; however, the main contributions are still present. On the contrary, only noise is present for Si@C. The spectra reappearance for Si@C and the decrease in the background noise in the spectra at 0.4 V and 0.8 V indicate that the SEI becomes thinner. This evolution is probably due to the “unhooking” of SEI pieces, as suggested by other studies, but could also be due to densification during delithiation.

Figure 8b presents the evolution of the O 1s spectra as a function of the voltage of the working electrode. Binding energies for typical compounds found in SEI are reported in Table 2. For both materials, the contribution of the C-O bonds is important and is attributed to the binder (CMC). Even if the quantity of SiO_x_ is more important for the Si material, the relative oxygen quantity in both silicon-based materials is similar to that of the “pristine” electrode, as evidenced by the two spectra similarities. The first evolution for both materials at 1.0 V (lithiation) concerns the C=O signal at 531 eV, which becomes more intense. This increase can be attributed to the decomposition of the FEC additive. Actually, after the first step, consisting of an F-loss, the resulting carbocation (with a C=O bond) can then polymerize [32]. The VC additive can also polymerize with almost the same mechanism as its structure is close to FEC and thus favors an increase in the overall quantity of C=O bonds in the SEI. Then, the decrease in the middle peak (pink) can also be attributed to the formation of inorganic compounds containing Li, coming from the decomposition of the electrolyte.

Important differences between both materials can be seen at 0.35 V (lithiation). For Si@C, the quantity of C = O bonds formed is more important as the peak attributed to this contribution (in orange) is more intense compared to the other peak of the spectra. Dedryvère et al. attributed this effect to the formation of organic species in the SEI [21]. Moreover, a peak appears on both spectra, centered at 528 eV. It corresponds to Li_2_O, whose presence in the SEI of Si-based anodes is mentioned as the final product of the decomposition of the Si oxides at the surface. This characteristic peak of Li_2_O can be observed on the following spectra (delithiation) for both materials. This compound was evidenced by Philippe et al. [38]. The authors demonstrated that Li_2_O was present at the surface of the material and its formation was reversible.

**Figure 9:**
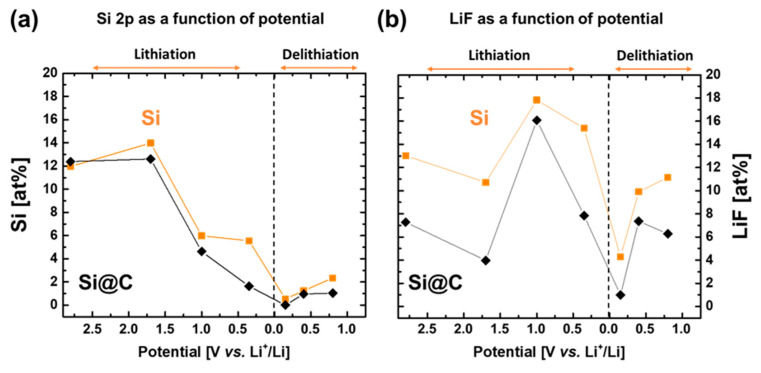
(**a**) Atomic percentages of Si in the total quantification of elements determined by XPS, giving indications of the SEI layer thickness on top of silicon surface; (**b**) atomic percentages of lithium fluoride in the total quantification of elements determined by the peak surface area corresponding to LiF. LiF is the main inorganic component in the SEI.

Figure 9a presents the variation of the quantification of the Si 2p signal all along the first lithiation and delithiation. The contribution of the silicon signal (106 eV–99 eV), which is taken into account, is only the contribution of Si^0^_,_ as a reference of active material lying underneath the SEI. The decrease in the signal intensity can be interpreted as a thickening of the SEI layer since this signal is supposed to weaken as a consequence of the SEI deposition. It gives information on the SEI thickness evolution along the first cycle. For both materials, Si (orange) and Si@C (black), the global evolution of the atomic percentage, hence the thickness of the SEI, shows a constant deposition until the lowest voltage. During the delithiation, the SEI tends to slightly shrink as the Si 2p signal strengthens a little, which is a sign of a thinner SEI [38].

The specific evolution of the LiF quantity is presented in Figure 9b. Actually, LiF is the major inorganic product that is deposited during the formation of the SEI on the surface of silicon anodes [39]. LiF contribution appears in both the Li 1s and F 1s XPS spectra. The energy of this contribution is seen at 55.8 eV on the Li 1s signal and at 685 eV on the F 1s signal [37,40]. The quantification of LiF for those two contributions matches well as the atomic percentages of Li^+^ in LiF and F^-^ in LiF are close to 1:1. This evolution is quite similar for both materials, Si (orange) and Si@C (black). LiF deposition occurs even at OCP (before any voltage sweep) because of the highly reacting surfaces exhibited by the nanocomposites [39].

**Figure 10:**
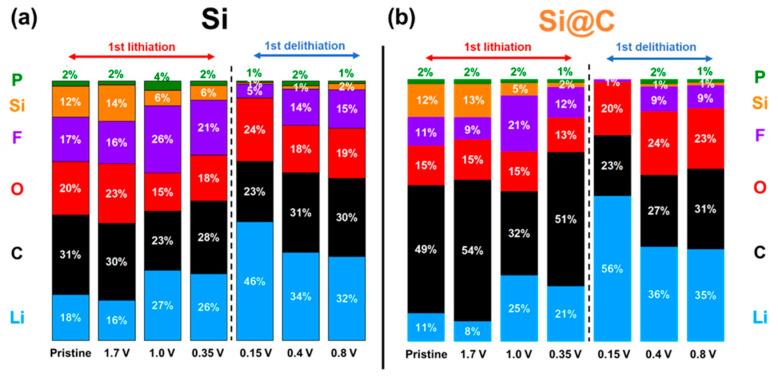
Quantification of each element analyzed for Si (**a**) and Si@C (**b**) materials during 1st cycle.

A complete quantification of the different scanned elements (Si, O, C, Li, F, P) is presented in Figure 10. It gives a global picture of the silicon-based electrode chemical surfaces evolution along the first cycle. Thus, it can be observed that, at a macroscopic level for the Si material, the surface tends to be covered with mainly LiF (between 1 and 0.35 V, lithiation), and then Li/O (0.15 V, delithiation), and then Li/F (>0.15 V, delithiation) elements, whereas, for the Si@C material, the SEI remains mostly based on organic degradation compounds (majority of C/Li elements), all along the cycle.

### 2.5. Comprehensive Discussion Coupling EIS and XPS

The chemical information from XPS can thus be compared with the former EIS data collected in order to better understand the SEI evolution that occurs at this early stage of cycling.

In “Zone I” (cf. Figure 5), the EIS measurements show two evolution steps in the SEI chemical composition for Si materials, correlated with an increase in the associated resistance. XPS quantification shows a more important quantity of LiF and Li_2_O as part of the SEI of the Si material. It seems that at low voltages, the nature of the SEI on Si materials is less conductive and more inorganic (due to the higher LiF amount and LiOx amount). In both materials, a thickening of the SEI occurs, as proved by the decrease in Si^0^ atomic percentage. However, in the case of the Si@C material, this SEI is probably conductive enough (being mostly organic) and thick enough to maintain the corresponding R_SEI,Si_ resistance at the same value (~0.2 Ω). During early delithiation stages (up to 0.3 V), both materials show a decrease in their respective SEI resistance (much bigger for Si nanoparticles), correlated with an XPS Si^0^ signal becoming more intense, consistent with a partial dissolution of the deposited SEI.

In “Zone II”, the EIS experiments show an increase in the resistance without any frequency change for the Si materials, which is consistent with a thicker or more densified SEI. Nevertheless, the increase in the atomic percentage of Si during the delithiation proves that the SEI becomes thinner. Thus, in “Zone II”, one hypothesis would be that the SEI during the delithiation process undergoes densification, inducing the increase by a factor of 9 of the associated R_SEI,Si_ resistance. By checking the corresponding SEI capacitance, the alpha coefficient of the relative CPE becomes closer to one, upon the delithiation process, which is a sign of a more homogeneous deposit, supporting the conclusion of such a densification mechanism.

## 3. Materials and Methods

### 3.1. Material Syntheses

The silicon-based materials presented here were synthesized by using a double-stage laser pyrolysis setup which has already been fully described by Sourice et al. [16,17]. In the first stage, a flow of silane gas precursor (SiH_4_) is perpendicularly intercepted by a CO_2_ laser (10.6 µm wavelength) in continuous mode. Silane is decomposed, which leads to silicon nanoparticles. Owing to an argon flow, silicon nanoparticles are pushed towards a second reaction chamber, where the flow meets ethylene gas (C_2_H_4_) and a second laser, leading to the deposition of a thin carbon shell around the pristine silicon nanoparticles. The resulting composite nanoparticles, Si@C, are finally pushed towards metallic filters, where they are collected as a dry phase, without using any solvent.

### 3.2. Electrochemical Characterizations

Electrodes are prepared by mixing the active material (50 wt%) with conductive “carbon Super P” (25 wt%), which ensures the electronic percolation and carboxymethylcellulose (CMC) binder (25 wt%), to obtain mechanically resistant electrodes. Materials are first introduced in distilled water with isopropanol as a wetting agent. The obtained liquid ink is then spread on a 10 µm copper foil with a 100 µm doctor blade. The foils are then stored in an autoclave overnight at 70 °C. Electrodes are then punched and calendered before being vacuum dried at 120 °C for 48 h. Electrodes are finally assembled, in a glove box, in two kinds of cells, one for galvanostatic cycling studies and one for the EIS studies, as described hereafter.

For galvanostatic studies, electrodes are assembled in a 2-electrode coin-cell configuration, with a lithium metal counter electrode. The electrolyte is a solution of LiPF6 at 1M in EC/DEC (vol. 1:1) containing 2 wt% vinylcarbonate (VC) and 10 wt% fluoroethylcarbonate (FEC).

For EIS studies, electrodes are assembled in a 3-electrode EL-Cell configuration, using lithium metal both as counter, and reference electrodes. The electrolyte used is the same as above. All the EIS measurements were performed after the cell reached relaxation which corresponds to a corresponding voltage drift inferior to 1 mV·h^−1^. The AC perturbation amplitude was 5 mV and the scanned frequency range was from 100 kHz to 0.1 Hz.

### 3.3. Analyses by Scanning Transmission Electron Microscopy–Electron Energy Loss Spectroscopy (STEM-EELS)

STEM–EELS experiments were conducted using an FEI Titan Ultimate microscope. The microscope was operating at an accelerating voltage of 200 kV. STEM–EELS maps were acquired using a Gatan GIF Quantum in the Dual EELS mode allowing the simultaneous collection of the low loss and core loss spectra.

### 3.4. Analyses by X-ray Photoelectron Spectroscopy

XPS was used to probe the chemical composition of the deposited SEI onto the particles of Si and Si@C during the first cycle. Electrodes are assembled in a coin-cell configuration. All the cells are then left untouched for twelve hours (relaxation). The cycling protocol was reproduced from the one used to compare Si and Si@C—19 wt% by EIS. The program consists in bringing the electrodes to the same period of time as the corresponding potential reached during the EIS measurements. A pristine electrode (wetted but uncycled) was also measured. Cells are then dismantled in glove box and the electrodes rinsed by short immersions in DMC before XPS analyses. A Versaprobe II spectrometer was used with Al monochromatized X-ray source. Spectra were calibrated relative to F 1s in LiF at 685 eV and O 1s at 528.5 eV for O in LiO_x_, the energy resolution was set at 0.6 eV with a pass energy of 23 eV (for high resolution spectra).

## 4. Conclusions

In this work, a general method to analyze SEI on silicon-based active materials through the combination of EIS and XPS measurements was presented. The first material is composed of nanoparticles with a silicon core of 30 nm diameter and the second one is composed of the same core, with a carbon coating. The better Coulombic efficiencies, which are observed in the case of Si@C, can be explained by the differences in SEI formation in the first cycles. In this paper, EIS and XPS were carried out to study the differences in the SEI composition and evolution at the first cycle. EIS experiments gave numerical values for both the resistance and the relaxation frequency of the SEI, leading to essential information about the thickness or the chemical composition of the layer during the first discharge and first charge. It was demonstrated that the SEI at the end of the first cycle on the Si particles is more resistive than the SEI on the Si@C particles. The specific composition of the SEI chemistry as a function of the presence of the carbon coating is most probably due to the different chemical nature of the active material surface, which is quite different in both cases (Si vs. C). The carbon coating, beyond the beneficial influence on the electronic percolation, offers another interface to the electrolyte, which may lead to a different interface chemistry. XPS has also demonstrated its utility, especially to reinforce the assumptions/conclusions from the EIS experiments. Our results demonstrated that the quantity of oxygen at the surface of the silicon was reduced in the case of carbon-coated silicon particles. The quantification of Si, C, O, P, Li, and F elements on the particle surface enabled the demonstration that the SEI on the Si@C particle contains more organic components.

The results show that the carbon coating in the studied core-shell Si@C nanoparticles acts as a protective layer for the silicon active material since the native silicon oxide layer is minimized. This paves the way towards processing this active material in mild conditions and is a promising step towards wider industrial developments of those materials. However, even if this work is a step forward in the understanding of SEI formation and chemistry, for such core-shell systems, more experiments could be carried out in the future. Ar^+^ sputtering XPS or Time-Of-Flight Secondary Ion Mass Spectrometry could be used, for example, to evaluate the 3D architecture of the SEI.

## Figures and Tables

**Table 1:** Chemical groups and their respective energies in the Si 2p signal. The references are also indicated.

Assignment	Binding Energy [ev]	References
Si-Si	~99.0–100.0	[25,26]
Si-C	100.3	[27]
SiO_x_	depends on *x*	[28]
SiO_2_	103.7	[29]
Si-O-C	101.3	[30]
O-Si-F	105.0–106.0	[22]

**Table 2:** Chemical groups and their respective energies in the O 1s signal. The references are also indicated.

Assignment	Binding Energy [ev]	References
Li-O in Li_2_O	528.5	[33]
Li-OH	531.9	[34,35]
C=O	531	[34,36]
C-OH	533	[34,36]
C-O	534	[33]
Si-O	532.6	[37]

## Data Availability

The data presented in this study are available on request from the corresponding author.

## Supplementary Materials

The following supporting information can be downloaded at: https://www.mdpi.com/article/10.3390/ma15227990/s1, Figure S1: (a) X-ray diagrams of the Si (orange) and Si@C (black) materials. The low quantity of SiC in Si@C leads to the morphology of a core-shell structure (b) Raman spectra of the two materials. The crystalline nature of Si is confirmed. The presence of C-C bonds is demonstrated by the D and G bands at 1350 cm-1 and 1600 cm-1.; Figure S2: (a) potential applied in function of the time during SPEIS analysis. The current response is indicated in blue (b) example of the potential (rouge) to current (blue) profile for the preparation of XPS samples. This shows the preparation procedure for the 0.35 V sample.
