# Supplementary material for: Electrochemical and X-ray Photoelectron Spectroscopic Study of Early SEI Formation and Evolution on Si and Si@C Nanoparticle-Based Electrodes
Source: Materials (Basel). 2022 Nov 11;15(22):7990. doi: 10.3390/ma15227990 (PMC9699462; doi:10.3390/ma15227990)
Supplement: Supplementary file 1 [file materials-15-07990-s001.zip › materials-1964639-supplementary.pdf]

Electrochemical and X-Ray photoelectron spectroscopicof early SEI formation and evolution on Si and Si@C nanoparticles-based electrodes

Antoine Desrues[1], Eric De Vito[2], Florent Boismain[1], John Alper[1], Cédric Haon[3], Nathalie Herlin-Boime[1] , Sylvain Franger[4]

1 : NIMBE, CEA, CNRS, Université Paris-Saclay, 91191 Gif-sur-Yvette, France

2 : Univ. Grenoble Alpes, CEA, Liten, DTNM, 38000 Grenoble, France

3 : Univ. Grenoble Alpes, CEA, Liten, DEHT, 38000 Grenoble, France

5 : ICMMO, UMR CNRS 8182, Université Paris-Saclay, 91405 Orsay, France

Supplementary Information:

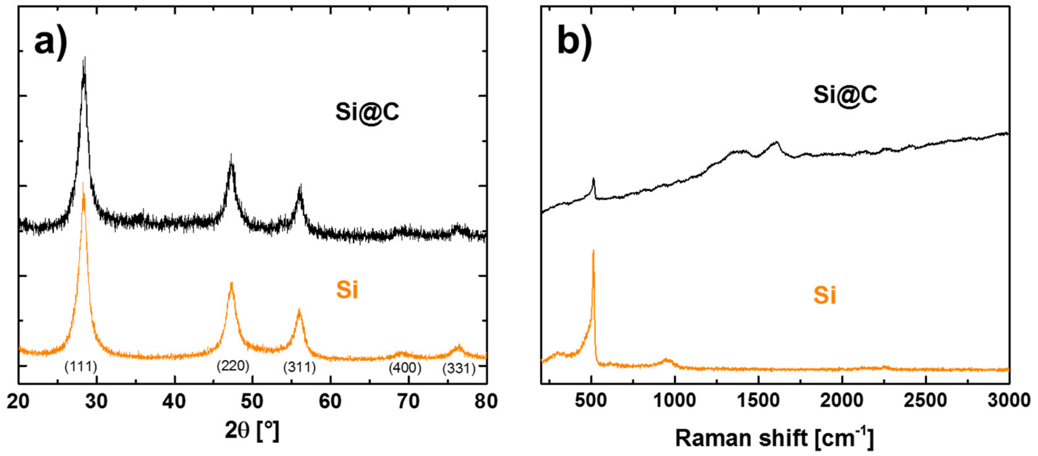

Figure S1 : (a) X-ray diagrams of the Si (orange) and Si@C (black) materials. The low quantity of SiC in Si@C leads to the morphology of a core-shell structure (b) Raman spectra of the two materials. The crystalline nature of Si is confirmed. The presence of C-C bonds is demonstrated by the D and G bands at 1350 cm^{-1} and 1600 cm^{-1}.

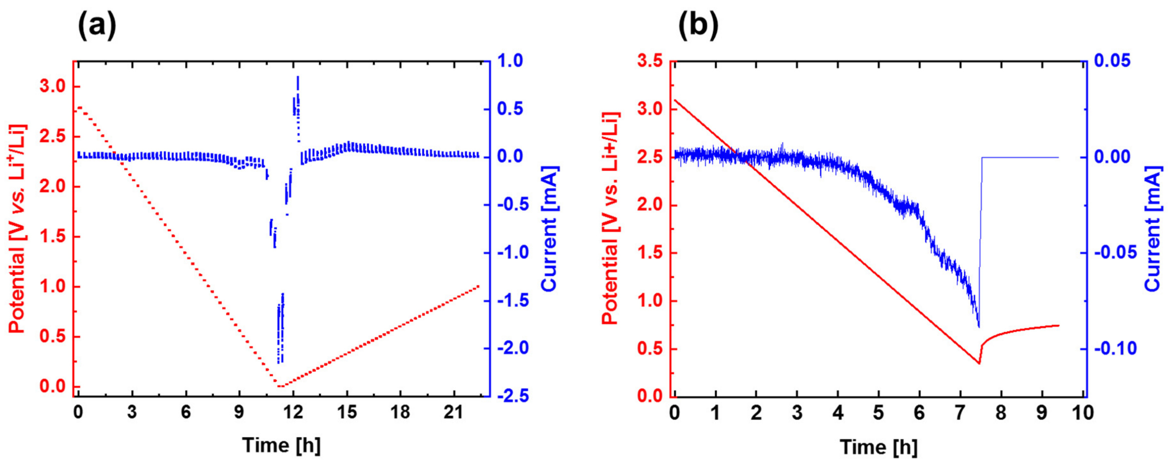

Figure S2 : (a) potential applied in function of the time during SPEIS analysis. The current response is indicated in blue (b) example of the potential (rouge) to current (blue) profile for the preparation of XPS samples. This shows the preparation procedure for the 0.35 V sample.
